# Spatial and temporal distribution of house infestation by *Triatoma infestans* in the Toro Toro municipality, Potosi, Bolivia

**DOI:** 10.1186/s13071-017-1984-0

**Published:** 2017-02-02

**Authors:** Jorge Espinoza Echeverria, Antonio Nogales Rodriguez, Mirko Rojas Cortez, Liléia Gonçalves Diotaiuti, David E. Gorla

**Affiliations:** 1 Laboratório de Referência Triatomíneos e Epidemiologia da Doença de Chagas, Centro de Pesquisas René Rachou, FIOCRUZ- MG, Belo Horizonte, Brazil; 2 Servicio Departamental de Salud - Potosi, Programa Nacional de Chagas, Potosi, Bolivia; 3 CEADES Salud y Medio Ambiente /Plataforma Chagas, Cochabamba, Bolivia; 4 Instituto Multidisciplinario de Biología Vegetal (IMBIV-CONICET), Córdoba, Argentina

**Keywords:** *Triatoma infestans*, House infestation, Chagas disease, Environmental variables

## Abstract

**Background:**

*Triatoma infestans* is the main vector of *Trypanosoma cruzi* in Bolivia. The species is present both in domestic and peridomestic structures of rural areas, and in wild ecotopes of the Andean valleys and the Great Chaco. The identification of areas persistently showing low and high house infestation by the vector is important for the management of vector control programs. This study aimed at analyzing the temporal and spatial distribution of house infestation by *T. infestans* in the Toro Toro municipality (Potosi, Bolivia) between 2009 and 2014, and its association with environmental variables.

**Methods:**

House infestation and *T. infestans* density were calculated from entomological surveys of houses in the study area, using a fixed-time effort sampling technique. The spatial heterogeneity of house infestation was evaluated using the SatScan statistic. Association between house infestation with Bioclim variables (Worldclim database) and altitude was analyzed using a generalized linear model (GLM) with a logit link. Model selection was based on the Akaike information criteria after eliminating collinearity between variables using the variable inflation factor. The final model was used to create a probability map of house infestation for the Toro Toro municipality.

**Results:**

A total of 73 communities and 16,489 house evaluation events were analyzed. Presence of *T. infestans* was recorded on 480 house evaluation events, giving an overall annual infestation of 2.9% during the studied period (range 1.5–5.4% in 2009 and 2012). Vector density remained at about 1.25 insects/ house. Infestation was highly aggregated in five clusters, including 11 communities. Relative risk of infestation within these clusters was 1.7–3.9 times the value for the regional average. Four environmental variables were identified as good descriptors of house infestation, explaining 57% of house infestation variability. The model allowed the estimation of a house infestation surface for the Toro Toro municipality.

**Conclusion:**

This study shows that residual and persistent populations of *T. infestans* maintain low house infestation, representing a potential risk for the transmission of *T. cruzi* in these communities, and it is possible to stratify house infestation using EV, and produce a risk map to guide the activities of vector control interventions in the municipality of Toro Toro (Potosi, Bolivia).

**Electronic supplementary material:**

The online version of this article (doi:10.1186/s13071-017-1984-0) contains supplementary material, which is available to authorized users.

## Background

Chagas disease remains one of the most important endemic diseases in the Americas [1]. The main mode of transmission in endemic areas is the vectorial route, by domestic, peridomestic, or sylvatic triatomines [2, 3]. At a global scale, an estimated 7 million people are infected by *Trypanosoma cruzi* [1]. In Latin America, the main vector is *Triatoma infestans*, a species widely distributed on the continent [4, 5], showing high vectorial capacity for the transmission of *T. cruzi*, because of its close association with man [6] and habitation of domestic and peridomestic structures [7].

The historical endemic area of Chagas disease in Bolivia covers around 60% of the country and corresponds to six of its nine Departments. Bolivia had one of the highest rates of house infestation by *T. infestans* and people infection by *T. cruzi* in Latin America [8–10]. The vectorial transmission of *T. cruzi* is almost exclusively due to domestic populations of *T. infestans* [11, 12], and (in some areas) this species was present in more than 98% of houses evaluated in rural and periphery-urban areas [13], before the implementation of the National Program of Chagas disease in the 2000’s. The Potosi Department, in the south of Bolivia, is considered one of the poorest (86% poverty), and has precarious housings that favor the establishment and development of triatomines (a scenario generally described in highly endemic areas of Chagas disease) [14–17]. These features are currently responsible for the persistence of *T. infestans* in 20 of the 40 provinces in the municipality of Toro Toro [18].

Within the first two decades after the initiation of the Southern Cone Initiative (INCOSUR) to control the transmission of *T. cruzi* by *T. infestans*, Brazil, Chile, Uruguay and wide areas of Argentina and Paraguay certified the interruption of the vectorial and blood-bank transmission of *T. cruzi* [19]. However, the success of the constant efforts to control *T. infestans* was limited in the Gran Chaco of Argentina, Bolivia and Paraguay and in some areas of the Inter-Andean Valleys of Bolivia [20, 21].

Traditionally, the control programs focus on the interruption of the vectorial transmission using insecticides with residual action (especially pyrethroids) to spray human dwellings and on the interruption of transfusional transmission of *T. cruzi* [22]. The main obstacle for a successful vector control intervention is the reinfestation of domestic and peridomestic structures from residual populations, that have either survived the insecticide treatment, or occupied houses that were not treated for some reason or from wild triatomine populations invading domestic premises [23–25]. Vector control failures due to high levels of insecticide resistance were reported mainly on the border between Argentina and Bolivia, in the biogeographical region of the Gran Chaco [26–28].

Identification of areas with persistent house infestation by the vector is very important for the control program management. The description and modelling of vector infestation distribution at the regional level are important inputs to understand the responses of the vector populations to a broad range of environmental conditions. This study aims at analyzing the spatial and temporal variation of house infestation by *T. infestans* in the municipality of Toro Toro (Potosi, Bolivia) during the period 2009–2014, and its association with environmental variables.

## Methods

### Study area

The study was carried out in the municipality of Toro Toro (Charcas Province), north of Potosi Department, Bolivia. It has an area of 1.172 km^2^, is inhabited by 12,086 people living in 3,511 houses in 73 communities [17]. Biogeographically, the area is part of the provinces of Puna and Bolivian-Tucuman [29], with an altitude ranging from 1,900–3,600 meters above sea level [30].

Farming is the main form of agriculture in Toro Toro, especially potato production and animal husbandry (cattle, goats, sheep and poultry). Of the total population, 97% are poor and 86% live in extreme poverty [31]. Housing is frequently precarious (81%). Most houses in the municipality are built with adobe, stone, thatched roofs and have dirt floors. Houses in the main town (Toro Toro) are of better quality. The majority of houses in the municipality has some peridomestic structure for the protection of domestic animals (i.e. goat corrals and chicken coops).

### House infestation and vector density

The information on house infestation by *T. infestans* was provided by the National Chagas Program of Bolivia (NPCCD), and Health Departmental Service (SEDES Potosi). Between 2009 and 2014 field teams evaluated house infestation in the municipality on an almost yearly basis. Although coverage was not complete, 16,489 house evaluation events were carried out during the period. A few communities were evaluated twice a year (Additional file 1: Table S1). The temporal analysis included estimates of the yearly house infestation recorded in the 73 communities in the municipality of Toro-Toro.

The annual entomological evaluation was carried out by field teams consisting of two technicians that spent 15 min searching inside houses (intradomicile) and peridomestic structures, completing 1-man-hour search on each house, according to standardized procedures of the Pan American Health Organization [32, 33].

House infestation (per year and for the whole study period) was calculated as the number of houses found to contain *T. infestans*, divided by the number of houses evaluated during the 2009–2014 period (Additional file 2: Table S2). Because houses were evaluated repeatedly during the study period, house infestation estimations might not be independent, and house evaluation within a community not always random. Because of this data feature, we carried out a detailed analysis of data properties (Additional file 3: Table S3), and decided the best option to estimate house infestation was to combine all infested and evaluated houses, by year. After the entomological evaluation, all infested houses were sprayed with alphacypermethrin at a nominal dose of 50 mg active ingredient/m^2^, using Hudson X-Pert™ manual sprayers. If house infestation at the community level was higher than 5%, all houses in the locality were sprayed, as indicated by the PAHO protocol [34, 35]. Annual house and community coverage was calculated as number of evaluated houses (or communities) divided by the number of houses in the community (or the number of communities in the municipality). *Triatoma infestans* density was calculated as the number of insects collected during the active search using the man-hour technique, divided by the number of infested houses.

### Data analyses

Data analysis focused on three main aspects of this study: description of house infestation and vector density during the period 2009–2014, detection of spatial heterogeneity of house infestation, and analysis of the association between house infestation and environmental variables.

Annual values of house infestation were calculated and risk of house infestation was compared using odds ratios (OR). Temporal trend in house infestation and vector density was analyzed using a generalized linear model (*glm* package of R 3.3.0) [36] and was compared using the Kruskal-Wallis test.

To analyze the spatial heterogeneity of house infestation, the spatial scan statistic [37], was used to measure possible spatial heterogeneity of house infestation and to detect clusters of significant high house infestation, compared with the regional average. The unit of analysis was the community, the circular shape of the spatial scan was used for cluster detection with a maximum circle size equal to 50% of the whole area, and the Poisson model was selected. Although data on house infestation were collected during 2009–2014, these were aggregated by community and the analysis was carried out as an atemporal, pure spatial process [38].

The association between house infestation by community and environmental variables was studied using a generalized linear model with a logit link. Environmental variables included the Bioclim set (Bio1 to Bio19) and altitude (i.e. 20 environmental variables) contained in the Worldclim database (http://www.worldclim.org/) [39] at 1 km^2^ spatial resolution. Bio1 to Bio9 include variables related to temperature, whereas Bio10 to Bio19 include variables related to rainfall.

Collinearity between environmental variables was estimated through the variance inflation factor (vif). Only environmental variables with vif < 10 (non-linearly related) were used to fit the final model. The model was used to produce a surface of estimated probability of house infestation in the study area using the *raster* package in R.

## Results

### Temporal and spatial analysis of house infestation and insect density

**Fig. 1:**
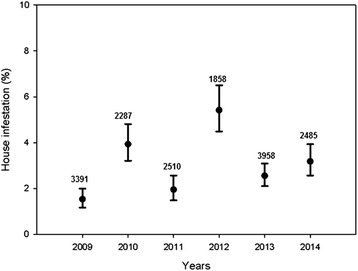
The annual average infestation in the municipality of Toro Toro (2009–2015). Vertical lines represent 95% confidence intervals. Numbers over vertical bars indicate the number of house evaluations during the year

House evaluation coverage within communities was relatively low, ranging from 30.8% (2012) to 65.6% (2013), whereas community coverage was higher, ranging between 45.2% (2011) and 80.8% (2009) during the study period (Table 1). A total of 16,489 house evaluation events were carried out over the 73 communities of Toro Toro from 2009 to 2014, giving an overall average of 0.78 house evaluations per house/year. Among these evaluation events, 480 gave positive results for the presence of *T. infestans*, giving an overall house infestation for the municipality of 2.9% during the studied period. Annual values of house infestation varied between 1.5% (2009) and 5.4% (2012) (Fig. 1). Compared with 2009 values, house infestation risk (measured by OR) was between 1.68 and 3.88 times higher in the following years, except in 2011, when infestation risk was similar to 2009 (Table 1).

**Table 1:** Community coverage (CC), house coverage (HC), number of house evaluations (HE) and house infestation (number of positive houses) by *T. infestans* in the study area between 2009 and 2014

Year	CC (%)	HC (%)	HE	No. of infested houses (%)	OR (95% CI)^a^	Density	
						ID	PD
2009	80.8	56.2	3,391	52 (1.5)	–	0.37	0.71
2010	65.7	37.9	2,287	90 (3.9)	2.63 (1.86–3.72)*	0.21	0.94
2011	45.2	41.6	2,510	49 (1.9)	1.28 (0.86–1.89)	0.67	2.53
2012	46.5	30.8	1,858	106 (5.4)	3.88 (2.77–5.44)*	0.24	1.56
2013	76.7	65.6	3,958	101 (2.5)	1.68 (1.19–2.36)*	0.04	1.65
2014	57.5	41.1	2,485	82 (3.2)	2.19 (1.54–3.11)*	0.04	1.38

*P < 0.05

^a^odds ratio, compared with infestation in 2009

*Abbreviations*: *CI* confinence interval, *ID* intradomestic, *PD* peri-domestic

Vector density varied between 1.1 and 3.2 insects per infested house (2009 and 2012, respectively). Although higher density was observed during 2011 and 2012, vector density remained at about 1.25 insects per infested house. Density in intradomestic structures was about 7 times lower (Kruskal-Wallis test, *χ* ^2^ = 50.75, *df* = 1, *P* < 0.0001) than density in peridomestic ones. Of the recorded infested houses, only 10% (48 out of 480) were colonized with nymphs, whereas the other harbored only adults.

**Fig. 2:**
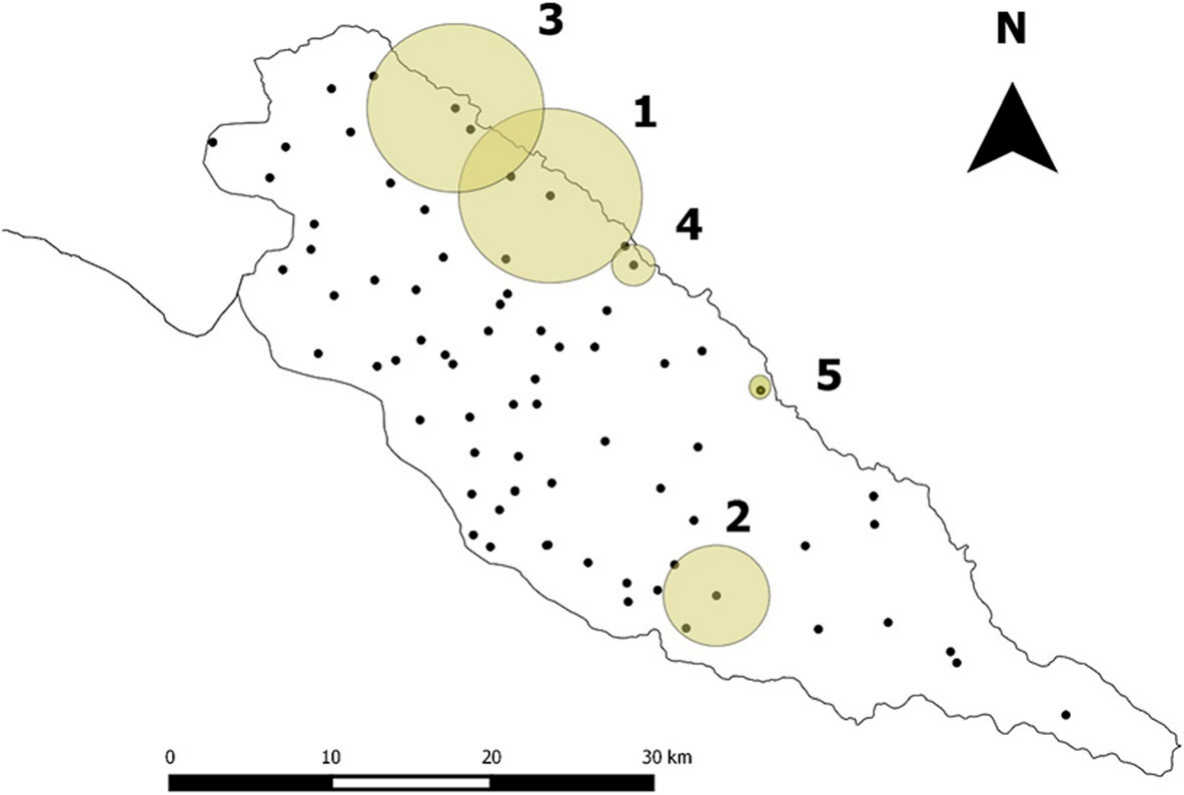
Distribution of the five clusters (*circles*) of high house infestation in the Municipality of Toro Toro. The size of the circles is proportional to the number of houses within each cluster. Black dots indicate the location of the rural communities included in the study

House infestation was not homogeneously or randomly distributed in Toro Toro, but showed an aggregated spatial distribution, with five significant community clusters of high house infestation (Fig. 2). These clusters grouped 4,359 of 16,489 house evaluation events from 11 of 73 communities in the municipality and showed values of house infestation between two and three times higher than the average house infestation (Table 2).

**Table 2:** High house infestation aggregates (clusters) in the rural communities of the municipality of Toro Toro

Clusters	Communities	Infestation % (CP/ CE)	Relative risk
1	Calahuta;	7.1 (131/1,852)	2.9
	Hacienda Loma;		
	Kehuayllani;		
	Sucusuma		
2	Omereque;	8.3 (46/566)	2.8
	Paloma Pampa		
3	Julo Chico;	4.9 (82/1,650)	1.8
	Julo Grande;		
	Kirusmayu		
4	Aguas Calientes	7.6 (30/390)	2.7
5	Quioma	7.1 (13/184)	2.6

*Abbreviations*: *CE* number of house evaluations, *CP* number of infested houses

### House infestation and environmental variables

**Fig. 3:**
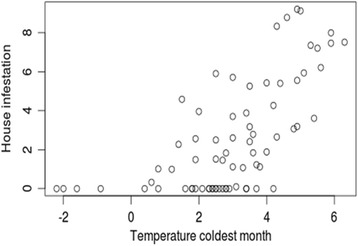
Dispersion diagram showing the relationship between the temperature (°C) of the coldest month and house infestation (%) in each rural community studied

**Fig. 4:**
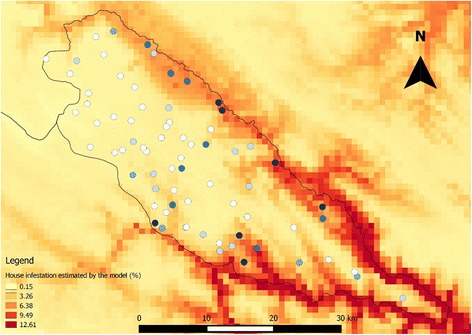
House infestation values estimated by the model with four Bioclim variables in the municipality of Toro Toro (see text). White to dark blue circles indicate the location of the communities and the observed house infestation throughout the study period (2009–2014), from low (*white circles*) to high (*deep blue circles*)

Four environmental variables proved to be good descriptors of house infestation in the study area. The glm produced a significant model that explained 57% of the variation, with an AIC of 391.58 with four environmental variables: mean diurnal temperature range (Bio2), temperature seasonality (Bio4), minimum temperature of the coldest month (Bio6) and precipitation of the coldest quarter (Bio19). A second model that only included the minimum temperature of the coldest month was also significant and showed as the second best model describing house infestation explaining 53% of the variation, with an AIC of 405.4. Although statistically different from the first model, this univariate model showed little difference of AIC and residual deviance values. No house was found infested in communities with temperature of the coldest month below 0 °C. Figure 3 shows the observed dispersion diagram between temperature of the coldest quarter and house infestation, whereas Table 3 reports on parameter estimates for both models. Using the estimated four-variable model mentioned above, and rasters of the four selected environmental variables, an estimated surface of house infestation was calculated for the municipality (Fig. 4). The estimated surface shows the spatial heterogeneity of house infestation, with higher values in the northern limit of the municipality, and along the southern hillsides and valleys towards the southeast.

**Table 3:** Estimated parameters of the generalized linear model for house infestation by *T. infestans* based on four environmental variables (*P* < 0.05). Null deviance: 497 on 72 degrees of freedom; residual deviance: 213.61 on 68 degrees of freedom; AIC: 391.58

Variable		Estimate	Standard Error	Pr(>|z|)
Intercept		-1.88	3.57	0.59
Bio 2	Mean diurnal temperature range	-0.04	0.02	0.05
Bio 4	Temperature seasonality	1.71e^-3^	4.0e^-3^	1.39e^-4^
Bio 6	Minimum temperature of the coldest month	0.03	7.11e^-3^	6.86e^-5^
Bio 19	Precipitation of the coldest quarter	-0.04	0.02	7.92e^-3^

## Discussion

Reports on house infestation by triatomines in Bolivia date back to pre-Columbian times [11, 40]. Indices reported in rural areas (70–100%) were higher than those reported in peri-urban areas (40–60%) [41, 42]. From 2000 onwards, a considerable reduction of house infestation by *T. infestans* was attained in Bolivia. However, only the Departments of La Paz and Potosi were certified free of vectorial transmission [1, 18], although the species persists in reduced population remnants throughout these Departments.

Within highly endemic provinces in the Department of Potosi, the municipality of Toro Toro (Charcas Province) draws attention due to the extreme poverty in the region [18, 31] and precarious housing, which generally favor the establishment and development of domestic triatomines. Such a scenario is typical in highly endemic areas [14, 15]. Before the intervention of the vector control program in 2000, the municipalities of the Potosi Department showed house infestation of up to 75%, which can be considered high when compared to current estimations [18].

In this study, we estimated that on average, the domestic infestation between 2009 and 2014, in 73 communities in the municipality of Toro Toro was 2.9%, similar to domestic infestation observed in the neighbor Department of Cochabamba between 2004 and 2011, where after a chemical intervention, the domestic infestation indices were 2–3% [43]. In other areas, outside the Andean valleys, within the Bolivian Chaco, the household infestation after intervention with insecticides dropped to 1.7% [44].

Comparing house infestation at the beginning of the study period (2009), the risk was always higher in subsequent years (odds ratios between 1.68 and 3.88), except for 2011. The lowest and highest value of annual infestation in the Toro Toro municipality were observed in 2009 and 2012. This variation could be the result of differences in the number of communities covered by the control program (80.2% in 2009 and 46.5% in 2012), and probably because in these communities only 56.2 and 30.8% of the houses were evaluated each year.

Despite the great effort by the vector control program, it is not always possible to cover all of the communities or houses. Low coverage can be attributed to the difficulties accessing certain communities, the absence of owners at the time of the evaluation, or other high priority endemic diseases draining personnel and/or funding. Beside low coverage, vector control efficacy could also be related to the vectors low susceptibility to pyrethroids. Although variable, house infestation by *T. infestans* in Toro Toro remains low (an overall 2.9%), but similar to those for houses in the neighbor regions of Potosi and Cochabamba.

It is important to emphasize that house infestation indices in the municipality before the establishment of the vector control program were as high as 80% [18]. As a consequence, high prevalence rates of Chagas disease were recorded in different age groups of the affected communities [12]. According to our results, house infestation remains low; however, a latent risk of *T. cruzi* transmission remains in these communities due to the persistence of *T. infestans* in both intra- and peridomestic structures. As such, it is important to maintain up-to-date knowledge of vector numbers, the cause(s) of the persistence of the house infestation, and control activities.

The low density of *T. infestans* in domestic environments observed during this study may be due to house construction material(s) [45, 46], the size of the dwelling and the number of individuals per room, the presence of domestic animals [47–49] and the use of household insecticides [46]. Higher densities of triatomines observed in the peridomicile may be due to the numerous structures (i.e. goat corrals and chicken coops) that create favorable microclimatic conditions for *T. infestans* [50, 51] by providing refuges and oviposition sites [46, 48], and refuges from insecticides.

The pattern of house infestation within the studied communities was highly clumped, heterogeneously distributed throughout the municipality of Toro Toro. The infestation clusters including 11 communities showed house infestation ranging from 7 to 8%. The spatial analysis of house infestation by *T. infestans*, as shown here, helps in the stratification of risk in rural communities of endemic areas. The identification of risk factors and spatial heterogeneity over wide geographical areas [52, 53], as used in studies of various disease vectors, may help to improve the efficacy and efficiency of vector control program [54–56].

The identified clusters of high house infestation could encompass high risk transmission areas, and should receive special consideration, and possibly more resources, by the vector control program. The relatively high infestation indices recorded in these geographical clusters could be due to various factors such as poverty, economic activities performed to sustain the household (animal husbandry), and local farming habits (special arrangement of peridomestic ecotopes, such as chicken coops and corrals). Low educational level of the population, unawareness of the vector presence risk, the efficacy of the vector control interventions, environmental factors, and potentially the presence of pyrethroid-resistant populations could also account for the observed outcome. According to the observations by Porcasi et al. [57], a number of these factors played an important role in the presence and extension of the high infestation clusters in the Argentine Chaco region. Similar factors could be contributing to the persistence and house infestation by *T. infestans* in the studied communities within Toro Toro.

The variables of the Bioclim dataset allowed the characterization of habitats within the environmental hiperspace inhabited by a species [58] and are commonly used to identify, and predict insect distribution areas for individual species [59]. The environmental analysis showed that two alternative models are able to describe the house infestation in the area. The best of the two models explained 57% of the variation, with four variables: two related to temperature and two to precipitation. The second model explained 53% of the variation, with only one variable, the minimum temperature of the coldest month, a similar finding to the one reported for the geographic distribution of *T. infestans* in the southern cone of South America [52].

## Conclusions

This study shows the importance of spatial analysis of the persistence of house infestation by *T. infestans* that obliges the maintenance of the vector vigilance and control activities, as well as the identification for the causes of the persistence of this house infestation. The effort to maintain vigilance and control could be efficiently organized following the results reported in this study that helps to spatially stratify the transmission risk over the communities of Toro Toro. This risk stratification could guide the differential assignment of resources by the public health agency of Potosi.

## Additional files

Additional file 1: Table S1. Number of localities evaluated once or twice a year during the study period. (XLSX 22 kb)
Additional file 2: Table S2. Number of evaluated houses varied within and between years. (XLS 19 kb)
Additional file 3: Table S3. Number of evaluated and infested houses according to each of the three methods described above. (XLS 22 kb)
Additional file 4: Figure S1. House infestation estimated by four alternative methods, based on different assumptions (see text). Blue rectangle: all yearly sample data aggregation, red diamond: highest yearly sample size, yellow triangle: maximum yearly infestation sample, green triangle: first yearly evaluation. (TIF 5074 kb)
Additional file 5: Supporting Information (DOC 32 kb)

## Abbreviations

CC: Community coverage
CE: Number of house evaluations
CI: Confidence interval
CP: Number of infested houses
EV: Environmental variables
GLM: Generalized linear model
HC: House coverage
HE: House evaluations
HI: House infestation
ID: Intradomestic
NPCCD: National Chagas Programme
ns: Not significant
OR: Odds ratio
PAHO: Pan American Health Organization
PD: Peridomestic
SE: Standard error
SEDES: Health Departmental Service
vif: Variance inflation factor
