# Supplementary figures and images for: Spatial and temporal distribution of house infestation by Triatoma infestans in the Toro Toro municipality, Potosi, Bolivia
Source: Parasit Vectors. 2017 Feb 2;10:58. doi: 10.1186/s13071-017-1984-0 (PMC5288887; doi:10.1186/s13071-017-1984-0)

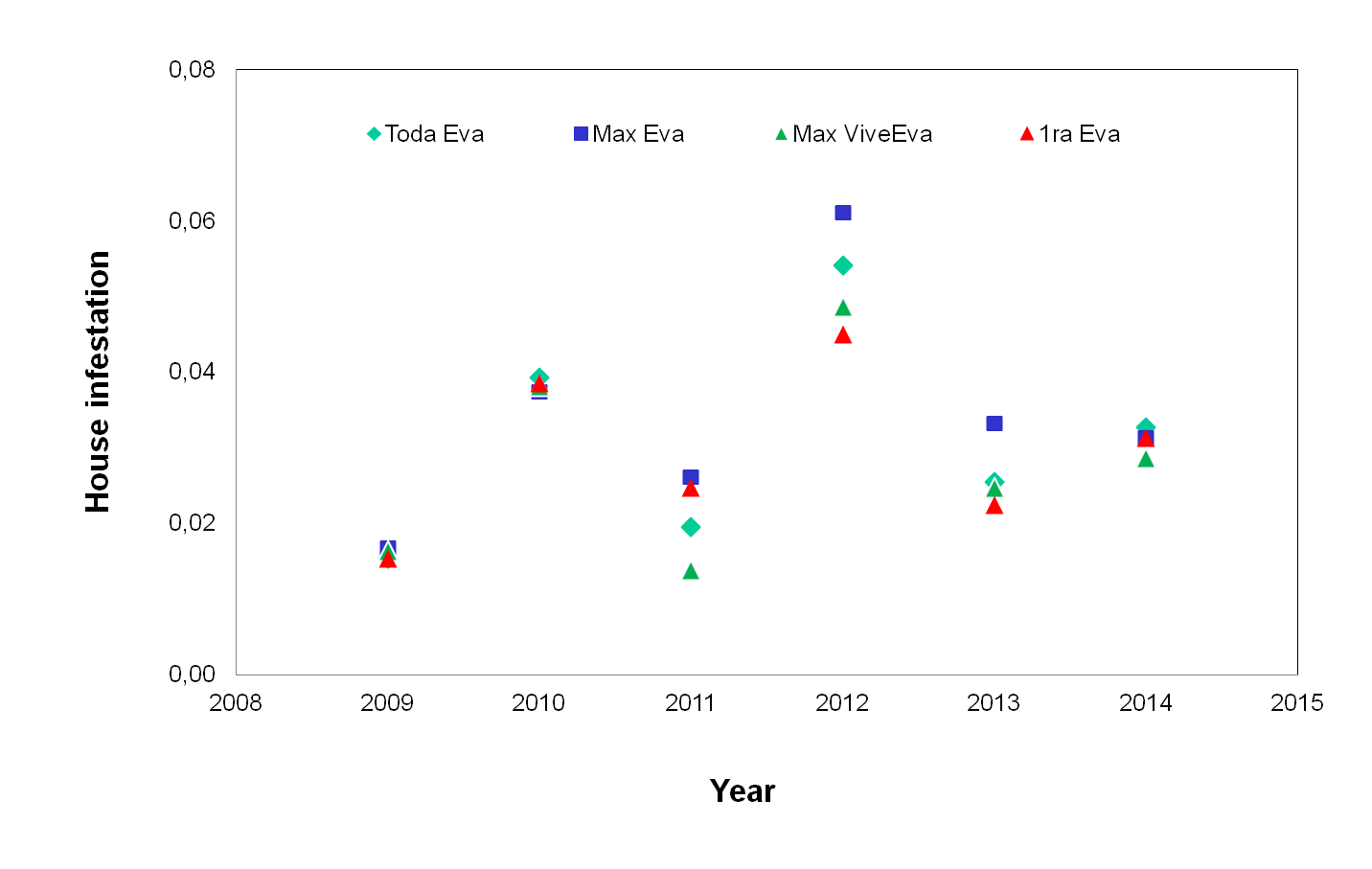

Supplement: Additional file 4: Figure S1. — House infestation estimated by four alternative methods, based on different assumptions (see text). Blue rectangle: all yearly sample data aggregation, red diamond: highest yearly sample size, yellow triangle: maximum yearly infestation sample, green triangle: first yearly evaluation. (TIF 5074 kb) [file 13071_2017_1984_MOESM4_ESM.tif]
