# Supplementary material for: Spatial and temporal distribution of house infestation by Triatoma infestans in the Toro Toro municipality, Potosi, Bolivia
Source: Parasit Vectors. 2017 Feb 2;10:58. doi: 10.1186/s13071-017-1984-0 (PMC5288887; doi:10.1186/s13071-017-1984-0)
Supplement: Additional file 5: — Supporting Information (DOC 32 kb) [file 13071_2017_1984_MOESM5_ESM.doc]

**Supporting Information**

**Estimation of house infestation**

House infestation is defined as the fraction of houses infested by the triatomine vector, i.e. number of infested houses/ number of evaluated houses (PAHO protocol) (WHO, 2006).

The analysis of this quantity in longitudinal studies has a number of caveats. The standard surveillance protocol for *T. infestans* infestation of rural houses in Bolivia implies the evaluation of each house twice a year by technicians from the local health agency, and the application of insecticide in the infested house and nearby neighbors. For a longitudinal study (6 years in the case of the present study), it was argued that the data analysis should take into account the non-independence of observations because the entomological status of a house in one moment would affect its entomological status in the following evaluation (Espinoza et al. 2014). This might be true, or not. A positive (infested by *T. infestans*) house in t might indicate a higher chance of finding it positive in t+1, perhaps because the house structure and cultural habits of owners could promote the infestation by *T. infestans*. However, a positive house in t should promote the application of insecticide and hence it should be found negative in t+1, as the consequence of the insecticide effect.

To evaluate the independence of consecutive entomological evaluations of houses included in the present study, we fit regressions for house infestation (= fraction of infested houses) in t vs house infestation in t+1, and for the number of infested houses in t vs number of infested houses in t+1. We included house infestation and number of infested houses as the former indicates the entomological status of houses in a locality, independent of the sample size, although strongly affected for small sample sizes. We included number of infested houses because this indicator is not affected by small sample size, although the dependent variable (y) can never have a value higher than the corresponding x value.

We carried out regressions for consecutive years between 2009 to 2013 of a linear type for house infestation and generalized linear regression with a quasipoisson error distribution (because of overdispersion detected using a Poisson distribution). All 5 linear functions fit for house infestation showed non significative slopes. Function fits for number of infested houses in 2011-2012, 2012-2013 and 2013-2014 showed non significative slopes, and significant ones for 2009-2010 and 2010-2011 (Table S1). In the latter case, slope value, although significative, was rather low (0.165). Considering these results, we find no strong evidence of temporal correlation between consecutive entomological evaluation. Additionally, more important than temporal correlation, is the question of house sampling in the localities, as we show below.

The protocol for the entomological evaluation of the Programa Nacional Chagas Bolivia indicates that every house of a locality in an endemic region should be evaluated every 6 months. This target is infrequently achieved. For a number of reasons, not all houses are reached on a sampling occasion and frequently, a second house evaluation in a year is used to evaluate houses not evaluated during the first one, and/or re-evaluate houses that were positive n the previous evaluation. Although some triatomine control programs individually identify each house (eg 37, 51), this is not the case for the Programa Chagas Bolivia and as a consequence, of the evaluated houses on one occasion we do not know wether there was a repeated evaluation on a following evaluation. This situation is a clear indication that houses are not sampled randomly, and this has probably a more serious effect on the estimation of house infestation. As house infestation is a key indicator for our study, we explored different methods for its estimation, as we explain below.

**Table S2** shows that the number of evaluated houses varied within and between years. We do not know wether within one year a house evaluated in a second evaluation has been or has not been evaluated during the first evaluation. We explored four alternative methods for the calculation of house infestation. In the first method, we took the evaluated houses (and the corresponding infested ones) on the occasion when the sample size was greater than other occasion within a year, assuming that a greater sample size would be better for the estimation of house infestation. In the second occasion, we took the houses that were evaluated during the first evaluation of the year, assuming that during this first evaluation, field teams would target all houses within the locality, whereas they would target previously positive houses (repeated evaluation) on the second yearly evaluation. In the third method, we took house infestation as the highest value of house infestation within each year, accepting this would represent a overestimation of house infestation. In the fourth method we added all evaluated houses, accepting there would be some error because of the repeated evaluation of houses, although using all collected data that eventually included all houses within a locality (either on the first or on the second evaluation).

**Table S3** shows the number of evaluated and infested houses according to each of the three methods described above. Fig S1 shows that estimation of house infestation is very consistent and independent of the method used. After this evidence, we decided to use the fourth method to estimate house infestation, eg. aggregating all houses evaluated and infested for each year.

**Fig. S1:** House infestation estimated by four alternative methods, based on different assumptions (see text). Blue rectangle: all yearly sample data aggregation, red diamond: highest yearly sample size, yellow triangle: maximum yearly infestation sample, green triangle: first yearly evaluation.
